# Inter-regional patient outmigration to Seoul in South Korea: the role of regional healthcare quality perceptions

**DOI:** 10.1186/s12913-025-12464-8

**Published:** 2025-03-19

**Authors:** Moo Hyuk Lee, Ji-Su Lee, Young Kyung Do

**Affiliations:** 1 Department of Health Policy and Management, Seoul National University College of Medicine, Seoul, South Korea; 2 Graduate School of Data Science, Korea Advanced Institute of Science and Technology, Daejeon, South Korea; 3 Institute of Health Policy and Management, Seoul National University Medical Research Center, Seoul, South Korea

**Keywords:** Perceived quality, Feeling of reassurance, Outmigration, Hospital choice, Regional healthcare system

## Abstract

**Background:**

Public perception of healthcare quality reflects a people-centered approach to evaluating quality and influences healthcare utilization. Patient choice of healthcare providers is not solely based on objective measures, but varies with perceived quality factors such as experiences and trust. In South Korea, a large number of patients with severe diseases bypass their regional tertiary hospitals and receive treatment from a few tertiary hospitals located in the capital city Seoul: that is, they outmigrate. In this paper, we aimed to directly measure the public’s feeling of reassurance with their regional healthcare system and examine it in explaining patient outmigration in South Korea.

**Methods:**

The data of this study came from an online survey involving 1,241 individuals that was conducted in 2020 − 2021 to investigate healthcare-related perceptions of the public. Using stated preference data on hypothetical vignettes involving a cancer diagnosis, we measured outmigration and feeling of reassurance. We performed a logistic regression to assess the association between the two variables, controlling for tertiary hospital beds, distance to Seoul, and sociodemographic characteristics.

**Results:**

Among 581 respondents, 65.6% reported that there is a regional hospital they felt reassured to visit when diagnosed with cancer, while 63.5% were inclined towards outmigration to Seoul when they need surgery for lung cancer. There was a clear and robust negative association between outmigration and feeling of reassurance, where individuals who felt reassured with their regional healthcare system were 18.6% points less likely to outmigrate to Seoul.

**Conclusions:**

Individuals’ feeling of reassurance with the regional healthcare system plays a crucial role in outmigration in South Korea. These results emphasize the need to consider patients’ subjective perception of quality in analyzing patients’ decision-making and hospital choice. Policy efforts to alleviate the concentration of patients into Seoul should consider how the public perceives and interprets the regional-level quality of care.

**Supplementary Information:**

The online version contains supplementary material available at 10.1186/s12913-025-12464-8.

## Background

Public perception of healthcare quality is important not only because it reflects a people-centered approach to evaluating quality but also because it influences healthcare utilization, thereby continuously reshaping the health system. Patient perceptions of the quality of healthcare services act as a key driver of patient choice of healthcare providers and other behaviors [1], a growing field of interest in the healthcare sector [2]. When patients choose which hospitals to visit, they do not always select the nearest hospitals; nor do they simply rely on objective measures of quality, such as healthcare resources and treatment outcomes [2, 3] – rather, their choice varies with diverse individual characteristics as well as their perception of healthcare quality. While previous studies have shown that patients’ choice of hospitals is affected by various factors such as individual sociodemographic characteristics, healthcare resources, hospital waiting time, procedure volume, and treatment outcomes [2–11], these observable factors alone do not fully account for the decision-making process. Perception of healthcare quality and subsequent hospital choice may also be affected by a range of less visible factors such as previous patient experiences, recommendations from families or friends, hospital reputation, trust and other quality indicators [2, 12–16]. Notably, a recent paper by Conlan et al. [17] highlights the unique features of perceived quality and its implications for health policy, and proposes a methodology to quantify this important conceptual construct.

The bypassing behavior – where patients choose not to visit the nearest local hospitals but to travel further to receive care – can shed light on the importance of perceived quality as a result of cumulative interactions between patients and local healthcare providers [4, 18]. In South Korea, a large number of patients with severe diseases bypass their regional tertiary hospitals and receive treatment from a few tertiary hospitals located in the capital city Seoul: that is, they *outmigrate*. Many patients hold the belief that several esteemed tertiary hospitals in Seoul provide substantially higher quality care for serious conditions such as cancer, backed by cutting-edge technologies and accumulated experiences [19, 20]. This perception persists despite the presence of tertiary hospitals in their own metropolitan cities and provinces that provide care within the same scope. The outmigration phenomenon has been aggravated in the past few decades due to the weak patient referral system and the development of transportation networks [20, 21], causing a multitude of problems to the overall healthcare system. If the quality gradient in tertiary hospital care between Seoul and other regions is real, systemic disparities in treatment access and outcomes can be exacerbated, assuming that outmigration may be disproportionately linked to individuals who possess socioeconomic advantages and can afford to do so. Patients who do outmigrate invariably shoulder a higher financial burden due to transportation and other expenses and often experience treatment delays, which can lead to even worse outcomes [22, 23]. Patient outmigration can also lead to underinvestment in non-Seoul regional healthcare systems and polarization in the allocation of healthcare resources between Seoul and other regions, which in turn may affect the public’s perceived quality of care and perpetuate the outmigration phenomenon [20]. The existence of this vicious cycle has necessitated policy efforts to mitigate the outmigration problem. It is crucial to understand the key drivers of patient outmigration to help policymakers explore effective solutions to this complex problem.

Previous studies and policy attempts in South Korea, however, have failed to go beyond examining individual or provider characteristics that are associated with patients’ decisions, without exploring perceived quality [24]. These studies found that outmigration was positively associated with income, surgery performance, living in provincial areas or areas without a metropolitan city, and negatively associated with distance to Seoul and combined comorbidity diseases [19, 23, 25]. While informative, such findings based on easily measurable proxies do not necessarily *explain* patient outmigration and often lead to policy discussions on increasing resources of provincial healthcare systems. From the perspective of patients and the public residing in non-Seoul regions, explanation for patient outmigration is rather straightforward and also in line with theory on patient choice of healthcare providers: what ultimately matters is the non-negligible difference in perceived quality of care between Seoul and their own region (all else being equal). Nevertheless, this explanation was not given sufficient attention.

To address this gap, we focused on the perceived quality of care and directly measured it to provide an explanation for patient outmigration in the South Korean context. To directly measure the public’s perceived quality of care in the regional healthcare system, we used the Sino-Korean word of *ansim* (안심, 安心), which can be loosely translated into English as *feeling of reassurance*. This word is not coined by the authors but frequently appears across a range of government policy briefs, press media, and popular discourse in South Korea, which suggests there is a shared (albeit implicit) understanding that patients’ perception of quality is critical in improving regional healthcare systems. Nevertheless, little research has been conducted on this concept as a causal factor in patient outmigration. This may be both in part because the public’s perception of care quality is not yet recognized widely as an academic topic that can and should be studied and in part because currently available data do not allow investigation for such a purpose. This study therefore aimed to directly measure the public’s feeling of reassurance with their regional healthcare system and examine it in explaining patient outmigration in South Korea.

## Methods

### Data

**Fig. 1:**
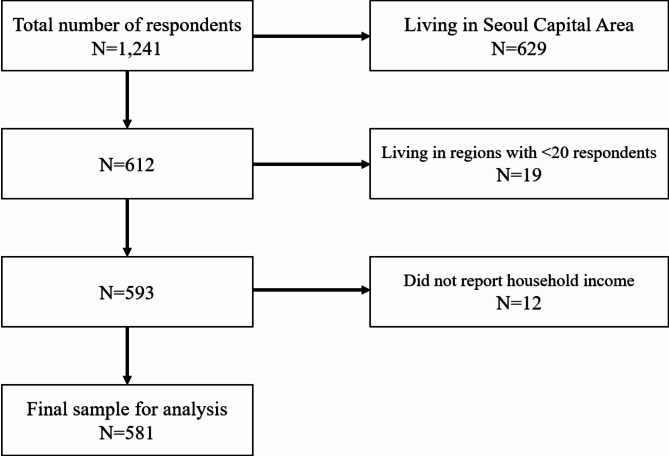
Flowchart of the exclusion criteria

We designed an online survey on a representative sample of the South Korean adult population (aged 19–69), with the primary aim to investigate the public’s opinions and viewpoints on healthcare utilization and their stated preferences for regional healthcare systems using hypothetical scenarios. Gallup Korea, a public opinion survey company, was entrusted with conducting the survey. A total of 1,241 individuals were included in the survey conducted between December 24, 2020, and January 5, 2021. Respondents were proportionally sampled based on residential area (17 regions consisting of 9 provinces and 8 special or metropolitan cities), gender, and age group (19–29, 30–39, 40–49, 50–59, and 60–69 years) [26]. Of 1,241 individuals who completed the survey, 629 respondents from the Seoul Capital Area (Seoul, Incheon, and Gyeonggi) were excluded from the study, as outmigration to Seoul applies only to individuals living outside the Seoul Capital Area. In addition, individuals living in two regions (Sejong and Jeju) with fewer than 20 respondents (*n* = 19) and those who did not report household income (*n* = 12) were also excluded, leaving our final sample of 581 respondents. We present the flowchart for the exclusion criteria in Fig. 1.

Additional data on the number of tertiary hospital beds were extracted from the Healthcare Big Data Hub provided by the Health Insurance Review and Assessment Service (http://gisopendata.hira.or.kr/). Although data acquisition was carried out in December 2021, the list of tertiary hospitals was based on December 2020 to maintain simultaneity with the survey data.

### Study variables

Our dependent variable, outmigration, was derived from responses on a hypothetical vignette of the respondent’s being diagnosed with lung cancer and needing surgery. We assigned 1 (= outmigration) if the individual would undergo surgery in one of the tertiary hospitals located in Seoul, and 0 otherwise.

The main explanatory variable of interest, feeling of reassurance, was defined using another hypothetical vignette. Respondents were presented with the scenario “Let’s assume that you were diagnosed with a severe disease such as cancer. Is there any hospital in your region (province or metropolitan city) which you feel reassured to visit?” We assigned 1 to those who reported “Yes” (vs. 0 to “No”). This survey question on feeling of reassurance was separated far away from the question on outmigration (Part 2 and Part 4, respectively) and was asked based on different hypothetical scenarios in order to avoid bias (An English translation of the original questions on outmigration and on feeling of reassurance is provided in Supplementary Materials 1).

Three sets of other explanatory variables were included in our regression model. First, we included the number of tertiary hospital beds, which was measured as the total number of beds in tertiary hospitals within a 1-hour travel distance from each individual’s residential area. We dichotomized the variable and assigned 1 for values above or equal to 3,800 beds (based on the median value 3,809), and 0 otherwise. Second, distance to Seoul was defined as a continuous variable of the travel time between each individual’s residential area and Seoul (with Seoul City Hall as the reference point). Travel time was calculated using the *osrmtime* command in Stata [27], which uses open-source routing machine (OSRM) data provided by OpenStreetMap (©OpenStreetMap Contributors, http://openstreetmap.org). Third, sociodemographic and health characteristics included gender, age, income, and whether the respondent has a chronic disease. Gender was a binary variable coded as 0 for males and 1 for females. Age was included as a set of five categorical variables (19–29, 30–39, 40–49, 50–59, and 60–69). Income was included as a set of 3-level categorical variables (low, middle, and high). Income was adjusted for different household sizes, by dividing the monthly household income by the square root of household size [26, 28]. The cutoff values for income were 2.12 million Korean Won (KRW) between low and middle, and 3.40 million KRW between middle and high. Finally, having at least one chronic disease was a binary variable coded as 1 for individuals with at least one chronic disease.

### Statistical analysis

We performed a bivariate analysis between the dependent variable (outmigration) and the main explanatory variable of interest (feeling of reassurance). Then, we conducted logistic regressions of outmigration, initially using each of the explanatory variables individually and subsequently using the complete set of the explanatory variables. All data analysis was performed using Stata SE 17.0 [29].

## Results

### Descriptive statistics

We present descriptive statistics of respondents in Table 1. Of the study sample of 581 respondents, 63.5% responded that they would receive surgery in a tertiary hospital located in Seoul (i.e., outmigrate), and 65.6% responded that there existed a regional hospital which they felt reassured to visit. The median value of tertiary hospital beds within 1 h was 3,809 beds, and the median distance to Seoul was 3.60 h. In terms of individual characteristics, 48.2% were female, median age was 47 years, the median adjusted monthly income was 2.71 million KRW, and 31.3% had at least one chronic disease.

**Table 1:** Descriptive statistics of the study sample

Variable	*N* (%)	Median (IQR)
**Outmigration**	369 (63.5%)	
**Feeling of reassurance**	381 (65.6%)	
**Tertiary hospital beds within 1 h**		3,809 (1,939, 5,146)
** Beds **$\:\ge\:$** 3**,**800**	299 (51.5%)	
**Distance to Seoul (hours)**		3.60 (2.19, 4.47)
**Female**	280 (48.2%)	
**Age (yr)**		47 (35, 57)
** Age group**		
** 19–29**	99 (17.0%)	
** 30–39**	99 (17.0%)	
** 40–49**	127 (21.9%)	
** 50–59**	139 (23.9%)	
** 60–69**	117 (20.1%)	
**Adjusted household income (million KRW)**		2.71 (1.90, 3.75)
** Income tertile**		
**Low (0 − 2.12)**	194 (33.4%)	
** Middle (2.13 − 3.39)**	196 (33.7%)	
** High (3.40−)**	191 (32.9%)	
**Having at least one chronic disease**	182 (31.3%)	

Percentages may not sum up to 100 because of rounding

*IQR* Inter-quartile range

### Bivariate analysis between outmigration and feeling of reassurance

We first explore the relationship between outmigration and feeling of reassurance (Table 2). The bivariate analysis yielded a statistically significant association (Pearson chi-square = 20.53, *p* < 0.001). Among individuals who reported that there is a hospital in their region they feel reassured to visit in case of severe disease such as cancer, 57.0% responded that they would visit a tertiary hospital located in Seoul, whereas the proportion of outmigration to Seoul increased by 19.0% points to 76.0% among those who answered negatively to the question of feeling of reassurance.

**Table 2:** Bivariate analysis of feeling reassured and outmigration

	Not feeling reassured	Feeling reassured
**No outmigration**	48 (24.0%)	164 (43.0%)
**Outmigration**	152 (76.0%)	217 (57.0%)
**Total**	200 (100.0%)	381 (100.0%)

Pearson$$\:{{\:X}}^{2}$$=20.53, *p* < 0.001

### Logistic regression

The first column of Table 3 shows unadjusted odds ratios (ORs) with their 95% confidence intervals (CIs) when a logistic regression model of outmigration was run on each of the explanatory variables individually. The unadjusted OR for feeling of reassurance was 0.42 (95% CI: 0.28, 0.61), indicating that feeling reassured with the regional healthcare system is associated with a substantially lower probability of outmigration. Having 3,800 or more tertiary hospital beds within 1-hour distance and a shorter distance to Seoul were each associated with a lower likelihood of outmigration.

In the full logistic regression results shown in the second column of Table 3, the adjusted OR for feeling of reassurance was 0.43 (95% CI: 0.29, 0.64), which is close to the corresponding unadjusted OR in magnitude. In terms of the marginal effect, this OR estimate translates to a probability difference of 18.6% points, which is similar to the result of the bivariate analysis presented above. Taken together, accounting for hospital resources and other covariates in the multiple logistic regression did not substantially alter the association between outmigration and feeling of reassurance. When feeling of reassurance is accounted for in the full logistic regression model, the number of tertiary hospital beds and distance to Seoul both decreased in terms of the strength of association, compared with their corresponding unadjusted ORs.

**Table 3:** Logistic regression of outmigration

Variable	Unadjusted OR (95% CI)	Adjusted OR (95% CI)
**Feeling of reassurance**	0.42** (0.28, 0.61)	0.43** (0.29, 0.64)
**Tertiary hospital beds ≥ 3**,**800**	0.68* (0.48, 0.96)	0.81 (0.56, 1.16)
**Distance to Seoul (hours)**	0.85* (0.73, 0.98)	0.90 (0.77, 1.05)
**Female (Reference: Male)**	1.24 (0.88, 1.74)	1.21 (0.85, 1.71)
**Age (Reference: 19–29)**		
** 30–39**	0.81 (0.45, 1.43)	0.81 (0.45, 1.47)
** 40–49**	1.03 (0.59, 1.79)	0.96 (0.54, 1.70)
** 50–59**	0.86 (0.50, 1.47)	0.84 (0.48, 1.46)
** 60–69**	1.09 (0.62, 1.92)	1.14 (0.64, 2.05)
**Adjusted household income (Reference: Low)**		
** Middle**	1.27 (0.84, 1.91)	1.33 (0.86, 2.04)
** High**	1.19 (0.79, 1.80)	1.35 (0.88, 2.07)
**Having at least one chronic disease**	0.95 (0.66, 1.36)	0.95 (0.65, 1.38)
***Constant***	─	4.05** (1.92, 8.57)
***N***	581	581

*OR * Odds Ratio, *CI * Confidence Interval

**p<0.001 *p<0.05

## Discussion

### Summary of the results and general implications

The key results of this study suggest that patients who reported they feel reassured with the regional healthcare system are less inclined to outmigrate to Seoul. This negative association was robust even after accounting for hospital resources, distance to Seoul, and other covariates in our analysis. Although this finding may seem obvious, no previous studies in South Korea have paid attention to this perspective of patients and the public. We aimed to address the existing gap in current research and policy debates, which almost exclusively rely on proxy measures of healthcare resources. Our further analysis using an additional survey question revealed that the majority (84.5%) of individuals who reported negatively to the question of feeling reassured with their regional healthcare system still affirmed the physical presence of local tertiary hospitals where they could receive treatment. This observation consolidates our premise that public perceptions of quality shaped by longstanding interactions between people and the regional healthcare system, rather than healthcare resources per se, may be a more important driver of the outmigration phenomenon and therefore a better causal explanation.

Our study contributes to existing knowledge by directly measuring perceived quality in a survey on the public. The variable of feeling of reassurance, which captures patients’ perceptions towards the regional healthcare system, is dynamic in that it accounts for longstanding interactions between patients and the health system and is prognostic in that potential patients reveal their expectations and potential decisions. Moreover, the association between outmigration and feeling of reassurance reflects the complex adaptive traits of the healthcare system, where non-linearity, unpredictability, policy resistance, phase transitions, and emergence are dominant [30–32]. Patients’ decisions to outmigrate likely stem from longstanding interactions within a complex network of patients and providers, in which the healthcare system plays a crucial role in determining each aspect of these network components.

Our findings are also closely related to the concept of health systems responsiveness, defined as “how well the health system meets the legitimate expectations of the population for the non-health enhancing aspects of the health system [33].” Initially proposed by the World Health Organization as one of the three intrinsic goals of health systems, responsiveness has increasingly been highlighted as a key dimension of overall performance assessment of the healthcare system [34, 35]. It includes initial expectations towards treatment as well as the experience arising from interaction between the people and the health system [36]. This patient-provider interaction is key to measuring responsiveness [37], since evaluation is performed based on people’s perception of what happens inside the interaction [33]. Such perception is created by a mix of individual experience, patient-provider interaction, beliefs, peer influence, and external information, which are core components of quality of care [31, 38]. This concept corresponds with the notion of feeling of reassurance directly measured in our study.

### Policy implications

From a policy viewpoint, our results suggest that policy efforts to alleviate the concentration of patients into Seoul should move beyond simply increasing hospital resources in non-Seoul regions and also focus on how the public perceives, understands, accepts, and interprets the regional-level quality of care. It is crucial to further investigate why (and why not) the public feels reassured with their regional healthcare system and to monitor effects of healthcare policies on the public’s perceived quality, possibly by conducting a routine population-based survey.

In particular, the impact of hospital bed availability on outmigration becomes statistically insignificant after adjustment. This finding suggests that resource allocation alone may not be sufficient to reduce inter-regional outmigration, nor should it be the sole focus for government policies aimed at mitigating outmigration. Instead, it highlights the need for active discussions on how to develop a regional healthcare system which provides reassurance to local residents, emphasizing ‘perceived’ quality over purely quantitative aspects. This aligns with a longstanding limitation of South Korea’s health policy, which has historically focused on quantitative expansion.

Another notable finding is that a substantial proportion of the respondents who felt reassured with the regional health system still chose to outmigrate. This phenomenon reflects the complexities of the South Korean healthcare system. One contributing factor is the high accessibility to Seoul, facilitated by extensive high-speed railway networks [21]. However, the underlying reasons are more deeply rooted in the healthcare system itself and arise from a complex interplay between patients, providers, and hospitals, forming multi-directional feedback loops. Outmigration once again exacerbates gaps in investment and hospital management, eventually discouraging physicians from working in non-Seoul regions and further widening both objective and perceived quality gaps [39].

### Limitations

Our study has several limitations that should be considered in future research. We first present limitations regarding the survey design. First, we used data of stated preference using a hypothetical vignette, instead of revealed preference. Stated preference and revealed preference differ from each other in that the former reflects expectations whereas the latter reflects experiences [40, 41]. Although expectations are formed based on experiences, the two are different entities and may provide different results. Our study focuses exclusively on patients’ expectations and the expected behavior arising from such expectations, which may not remain consistent in real-world data. Such revealed preference data should be analyzed to overcome such limitations in future research. Second, the hypothetical vignette of a diagnosis of lung cancer needing surgery employed in our survey is arguably a typical case of outmigration in South Korea but still might not fully represent the broader scenario of serious diseases experienced by non-Seoul residents. For more advanced policy discussions in the future, it would also be important to determine the appropriate level of concentration to tertiary or quaternary hospitals in Seoul, because concentration of patients, particularly with rare diseases in need of quaternary care, to high-volume hospitals leads to better outcomes in cases where volume-outcome relationship holds [42–44]. Third, our key explanatory variable, feeling of reassurance, was only measured as binary rather than continuous or at least ordinal, leading to an insufficient analysis of more subtle differences within the binary category. As an explorative and pioneering approach, we believe that treating the independent variable as a binary measure provides at least a preliminary understanding of the primary relationship between outmigration and reassurance. Relatedly, this variable did not undergo rigorous pretesting, although it was derived directly from policy discussions. Currently, we are developing a measurement instrument for reassurance (or health system responsiveness at local/regional levels) using primary data collected. Fourth, it may be argued that the main independent variable may be endogenous to the dependent variable. For example, it is plausible that individuals who are more likely to outmigrate are also those with lower levels of reassurance regarding their region’s healthcare quality (i.e., reverse causality). The use of instrumental variables could address this challenge; however, our data did not contain suitable instrumental variables that strongly predict individual-level feelings of reassurance without directly affecting outmigration.

Other limitations should also be noted regarding the conduction of the survey. First, the data collection period coincided with the COVID-19 pandemic, which may have influenced respondents’ preferences. While this survey measured stated rather than revealed preferences, respondents might have perceived the pandemic as a barrier to selecting certain hospitals or as a factor shaping their feelings of reassurance. It is likely that the impact of the ‘feeling of reassurance’ on ‘outmigration’ was underestimated, as respondents who did not feel reassured with the healthcare system might have been more inclined to outmigrate, had it not been for the constraints imposed by the pandemic. Second, the sample included only individuals aged 19–69, excluding patients 70 years and above. Older adults may have greater reliance on regional healthcare system, and face more complex medical problems as well as accessibility difficulties. Consequently, their exclusion may limit the generalizability of the findings. This limitation arose from practical challenges associated with conducting an online survey through a survey company. Third, the number of respondents in our final sample diminished considerably once we have excluded half of the survey respondents from the Seoul Capital Area, which accounts for roughly half of the South Korean population. This limitation precluded further detailed analysis. However, despite the exclusion of approximately half of the total respondents, we clarify that this did not affect the proportionality of the sample with respect to residential area, gender, and age group. A larger survey scale, perhaps with an oversampled subpopulation for provincial regions, would be beneficial in fully explaining variations in outmigration and its association with other potential predictors both at individual and regional levels.

Despite these limitations, our study contributes to the literature by directly measuring perceived quality and empirically demonstrating its negative association with patients’ decisions to outmigrate in South Korea. By explaining the outmigration phenomenon from the public’s perspective, our empirical results can better inform current policy debates to improve regional healthcare systems.

## Conclusions

Individuals’ feeling of reassurance with the regional healthcare system plays an important role in explaining outmigration in South Korea. Patients’ subjective perception of quality is crucial in analyzing their decision-making process and choice of hospitals. Policy efforts to mitigate the concentration of patients to Seoul should focus on how the public perceives the regional-level quality of care.

## Supplementary Information

Supplementary Material 1.

## Data Availability

In accordance with regulations and data privacy policies, the raw survey questionnaire and data used in this study cannot be publicly disclosed. The Health Insurance Review and Assessment Service (HIRA) possesses the right for the dataset. However, the Stata code used for data analysis is always available upon request, and a subset of survey questions used in analysis are provided in Supplementary Materials 1. Additionally, we can provide GIS data and detailed descriptions of its analysis, also available on request. Researchers interested in obtaining access to raw data may submit a formal request to HIRA according to their data access policies and procedures, but please note that final decision is subject to approval by HIRA.
