# Supplementary material for: Inter-regional patient outmigration to Seoul in South Korea: the role of regional healthcare quality perceptions
Source: BMC Health Serv Res. 2025 Mar 19;25:407. doi: 10.1186/s12913-025-12464-8 (PMC11921725; doi:10.1186/s12913-025-12464-8)
Supplement: Supplementary file 1 — Supplementary Material 1. [file 12913_2025_12464_MOESM1_ESM.pdf]

Supplementary Materials 1

Survey questions

We present the survey questions used for analysis, excluding the basic questions about sociodemographic characteristics. The original version was written in Korean.

Part 2: Perceptions on regional healthcare system

Let's assume that you were diagnosed with a severe disease such as cancer. Is there any hospital in your region (province or metropolitan city) which you feel reassured to visit?

1) Yes
2) No

Part 4: Healthcare utilization behavior (II): Hypothetical vignette

Let's consider the following situation: Your recent health examination revealed a lung nodule. The doctor at the hospital suspected a high possibility of cancer based on its shape and size. The doctor advised you to visit a nearby tertiary hospital in your region, and provided you with a referral letter for consultation.

(…)

Now let's assume that you received a tissue biopsy at a nearby tertiary hospital. The biopsy results confirmed a cancer diagnosis, and surgery is necessary.

In which hospital would you receive surgery, between the two options below?

1) A tertiary hospital located in your residential area
2) A large tertiary hospital in Seoul, equipped with a well-known surgeon for lung cancer surgery
